# Correction: Cross-cultural translation and psychometric validation of the French version of the Fear-Avoidance Components Scale (FACS)

**DOI:** 10.1371/journal.pone.0305371

**Published:** 2024-06-06

**Authors:** Arnaud Duport, Sonia Bédard, Catherine Raynauld, Martine Bordeleau, Randy Neblett, Frédéric Balg, Hervé Devanne, Guillaume Léonard

In Fig 2, there is an error in the fourth text bubble. It should have been “Excluded (n = 4) due to failure to respond to FACS a second time for test-retest, 7 days later”. The authors provided the correct version of Fig 2 here.

**Fig 2:**
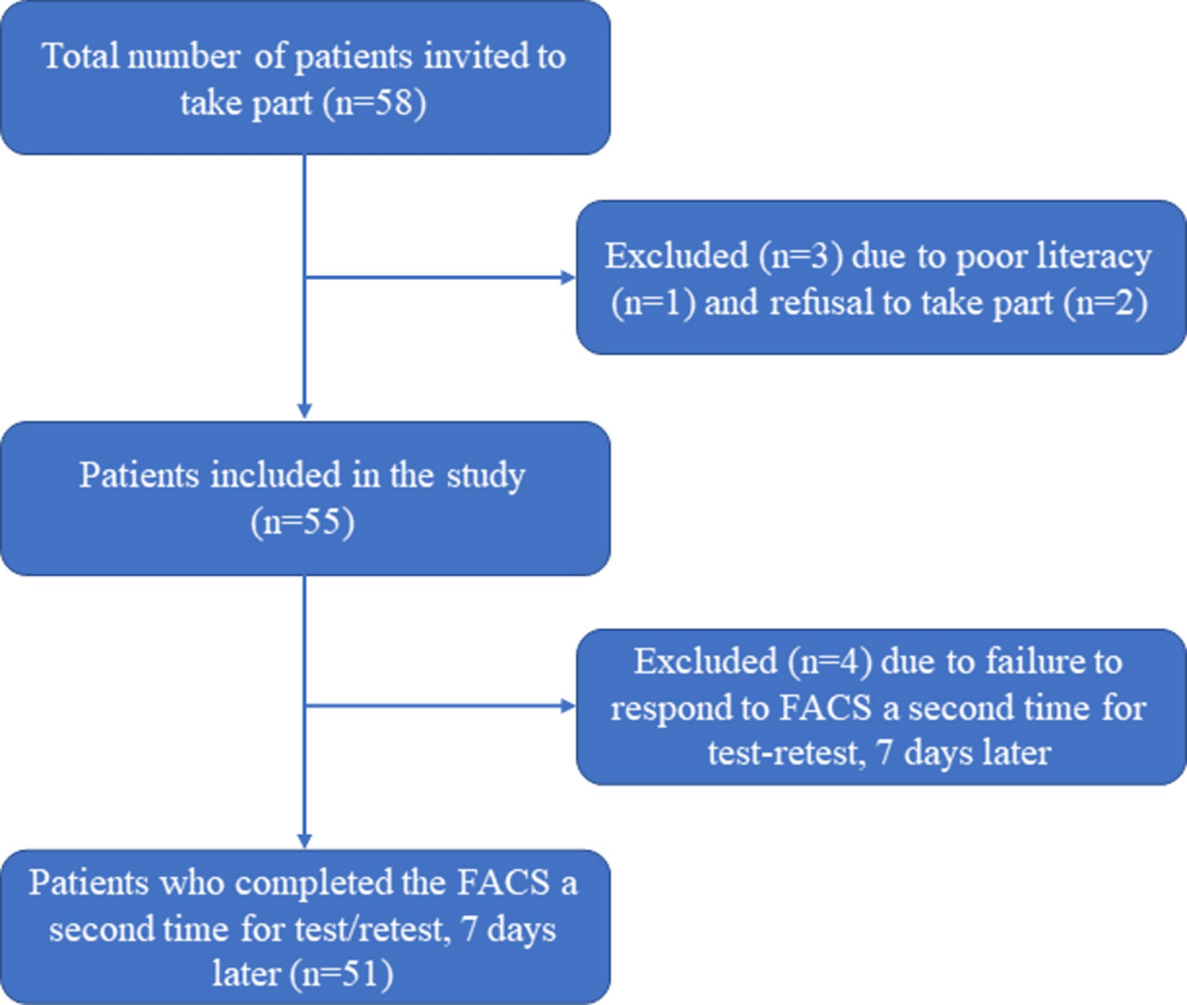
Patient recruitment flowchart.
