# Supplementary material for: Cross-cultural translation and psychometric validation of the French version of the Fear-Avoidance Components Scale (FACS)
Source: PLoS One. 2023 Oct 12;18(10):e0288899. doi: 10.1371/journal.pone.0288899 (PMC10569597; doi:10.1371/journal.pone.0288899)
Supplement: S1 File — (PDF) [file pone.0288899.s001.pdf]

FACS-Fr/CF

Nom :______________________________ ID # :_____________ Date :____/____/________

Instructions : Les gens réagissent à la douleur de différentes manières. Nous voulons savoir ce que vous pensez et ressentez à propos de votre condition douloureuse et comment elle a affecté votre niveau d'activité. Veuillez penser à votre état au cours de la dernière semaine et encercler un chiffre entre "0" et "5" dans l'échelle ci-dessous pour répondre à chaque question.

5 = Tout à fait d’accord

4 = Plutôt d’accord

3 = Légèrement d’accord

2 = Légèrement en désaccord

1 = Plutôt en désaccord

0 = Pas du tout d’accord

**Par rapport à la dernière semaine, dans quelle mesure êtes-vous en accord avec les énoncés suivants en ce qui concerne votre condition douloureuse ?**	Tout à fait d’accord	Plutôt d’accord	Légèrement d’accord	Légèrement en désaccord	Plutôt en désaccord	Pas du tout d’accord
1) J’essaie d’éviter les activités et les mouvements qui aggravent ma douleur…………………………………………	5	4	3	2	1	0
2) Je m’inquiète au sujet de ma condition douloureuse……	5	4	3	2	1	0
3) Je crois que ma douleur ne fera qu’empirer jusqu’à ce que je n’arrive plus du tout à fonctionner………………….	5	4	3	2	1	0
4) Je suis submergé(e) par la peur lorsque je pense à ma condition douloureuse……………………………………...	5	4	3	2	1	0
5) J’évite de pratiquer certaines activités, car j’ai peur de me blesser (ou de me blesser à nouveau)...………………...	5	4	3	2	1	0
6) Lorsque ma douleur est très intense, j’éprouve aussi d’autres symptômes comme des nausées, des difficultés respiratoires, des palpitations, des tremblements ou des étourdissements……………………………………….	5	4	3	2	1	0

Suite…

Par rapport à la dernière semaine, dans quelle mesure êtes-vous en accord avec les énoncés suivants en ce qui concerne votre condition douloureuse ?

	Tout à fait d'accord	Plutôt d'accord	Légèrement d'accord	Légèrement en désaccord	Plutôt en désaccord	Pas du tout d'accord
7) Il est injuste que je doive vivre avec ma condition douloureuse………………………………………………...	5	4	3	2	1	0
8) Ma condition douloureuse augmente le risque de me blesser (ou de me blesser à nouveau) pour le reste de ma vie………………………………………………………………..	5	4	3	2	1	0
9) En raison de ma condition douloureuse, ma vie ne sera plus jamais la même………………………………………..	5	4	3	2	1	0
10) Je n'ai aucun contrôle sur ma douleur…………………	5	4	3	2	1	0
11) J'évite certaines activités et certains mouvements, car j'ai peur que ma douleur augmente………………………...	5	4	3	2	1	0
12) C'est la faute de quelqu'un d'autre si j'ai cette condition douloureuse……………………………………...	5	4	3	2	1	0
13) La douleur causée par mon état de santé est un signal d'alerte indiquant que quelque chose ne va pas du tout……	5	4	3	2	1	0
14) Personne ne comprend à quel point ma condition douloureuse est grave………………………………………	5	4	3	2	1	0

Commencez chacune des phrases suivantes par ceci : Durant la semaine dernière, en raison de ma condition douloureuse, j'ai évité…

	Tout à fait d'accord	Plutôt d'accord	Légèrement d'accord	Légèrement en désaccord	Plutôt en désaccord	Pas du tout d'accord
15) …des activités exigeantes (p. ex., effectuer d'importants travaux d'entretien de terrain ou déplacer des meubles lourds)……………………………………...	5	4	3	2	1	0
16) …des activités modérées (p. ex., faire le repas ou le ménage de la maison).....……………………………………..	5	4	3	2	1	0

FACS-Fr/CF

Suite…

Commencez chacune des phrases suivantes par ceci : Durant la semaine dernière, en raison de ma condition douloureuse, j'ai évité…

	Tout à fait d'accord	Plutôt d'accord	Légèrement d'accord	Légèrement en désaccord	Plutôt en désaccord	Pas du tout d'accord
17) …des activités légères (p. ex., aller au cinéma ou au restaurant)......……………………………………….......	5	4	3	2	1	0
18) …l'ensemble de mes tâches et corvées à la maison et/ ou au travail…………………………………………...	5	4	3	2	1	0
19) …des activités récréatives et/ou de l'exercice (des choses que je fais pour le plaisir ou maintenir un bon état de santé)…………………………………………………	5	4	3	2	1	0
20) …des activités où je dois utiliser la ou les parties douloureuses de mon corps………………………………...	5	4	3	2	1	0

Score total : ____________________
